# FedSepsis: A Federated Multi-Modal Deep Learning-Based Internet of Medical Things Application for Early Detection of Sepsis from Electronic Health Records Using Raspberry Pi and Jetson Nano Devices

**DOI:** 10.3390/s23020970

**Published:** 2023-01-14

**Authors:** Mahbub Ul Alam, Rahim Rahmani

**Affiliations:** Department of Computer and Systems Sciences, Stockholm University, 16407 Stockholm, Sweden

**Keywords:** Internet of Medical Things, smart healthcare, clinical decision support system, deep learning, federated learning, multi-modality, natural language processing, electronic health records, early sepsis detection

## Abstract

The concept of the Internet of Medical Things brings a promising option to utilize various electronic health records stored in different medical devices and servers to create practical but secure clinical decision support systems. To achieve such a system, we need to focus on several aspects, most notably the usability aspect of deploying it using low-end devices. This study introduces one such application, namely *FedSepsis*, for the early detection of sepsis using electronic health records. We incorporate several cutting-edge deep learning techniques for the prediction and natural-language processing tasks. We also explore the multimodality aspect for the better use of electronic health records. A secure distributed machine learning mechanism is essential to building such a practical internet of medical things application. To address this, we analyze two federated learning techniques. Moreover, we use two different kinds of low-computational edge devices, namely Raspberry Pi and Jetson Nano, to address the challenges of using such a system in a practical setting and report the comparisons. We report several critical system-level information about the devices, namely CPU utilization, disk utilization, process CPU threads in use, process memory in use (non-swap), process memory available (non-swap), system memory utilization, temperature, and network traffic. We publish the prediction results with the evaluation metrics area under the receiver operating characteristic curve, the area under the precision–recall curve, and the *earliness* to predict sepsis in hours. Our results show that the performance is satisfactory, and with a moderate amount of devices, the federated learning setting results are similar to the single server-centric setting. Multimodality provides the best results compared to any single modality in the input features obtained from the electronic health records. Generative adversarial neural networks provide a clear superiority in handling the sparsity of electronic health records. Multimodality with the generative adversarial neural networks provides the best result: the area under the precision–recall curve is 96.55%, the area under the receiver operating characteristic curve is 99.35%, and *earliness* is 4.56 h. *FedSepsis* suggests that incorporating such a concept together with low-end computational devices could be beneficial for all the medical sector stakeholders and should be explored further.

## 1. Introduction

The traditional healthcare system was designed to ensure well-tested and intentioned efforts performed by expert professionals to improve or rehabilitate individuals’ physical and psychological health. Therefore, the most fundamental goal of the current medical approach is to give the resources to individuals to prevent or cure medically established diseases and disabilities [1]. The ever-expanding interconnectivity among different information generating electronic devices used by everyday people has provided us with the opportunity to help the healthcare system and medical facilities improve their overall aspects. We refer to this relatively new structural and usability paradigm shift as the Internet of Medical Things (IoMT). Healthcare professionals, patients, and other stakeholders can be interconnected through different electronic devices to exchange valuable related information with each other. Considering this, the structural (connection, communication, and information generation) and the usability (application) aspects are vital components in IoMT [2,3,4]. IoMT successfully utilizes the new wave of information explosion, the scale of inter-connectivity among electronic devices that we have never seen before, and the healthcare revolution transforming our notion of traditional healthcare systems. Effective prevention of diseases, patient-centric healthcare, real-time distance-based monitoring with automatic diagnosis support tools, improved collaboration among caregivers and patients, sustainable health and longevity, and low-cost healthcare for everyone are critical examples of IoMT applications [5]. We hope that this patient-centric approach will help reduce hospital costs, errors, and visit times. It will ensure effective distance-based management and remote monitoring of the patient’s health which will also help achieve sustainable development by reducing the urbanization trend as remote areas tend not to have better medical facilities. One of the keys to ensuring this is by employing effective clinical decision support systems to detect the disease using patient data early [6].

Clinical decision support systems (CDSSs) accommodate medical professionals in their decision-making process by providing intelligent and relevant information to diagnose, monitor, or manage patients’ health. A well-designed CDSS can enhance the overall performance by providing valid results, which should be convenient and confident in its prediction [7]. The notable examples of CDSS are disease detection/classification, sequential prediction of clinical events, concept embedding, data augmentation, and de-identification [8].

One way to ensure the excellent performance of a CDSS is by efficiently utilizing the medical data. IoMT can generate vast amounts of medical data. A crucial example of this type of data is electronic health records (EHRs). EHRs can be described as the digital records of all types of medical information of patients stored in different medical infrastructures. Therefore, it is ideal to utilize EHRs to create a robust CDSS using the IoMT setup. EHR can be extraordinarily sparse and heterogeneous, consisting of prescription notes about medicines; diagnosis history; clinical text from doctors and nurses; image-based, numeric, and categorical information from body vitals and laboratory tests, and demographic information about the patient [9,10]. This exceptional heterogeneity of the data and sensitivity of the information introduces some fascinating but challenging problems to consider before utilizing it in a CDSS. The effective utilization of EHRs should ensure *improved predictability* in terms of diagnosis. The *heterogeneity* aspect should also be handled carefully to obtain the optimum performance from the CDSS. The data should be explored with the utmost *security and privacy* to avoid information breaches.

Machine learning (ML) [11] is a solid choice to ensure *improved predictability* in CDSSs; which is a compendium of probabilistic computational methods to predict a decision using data for a particular task. Usually, several parameter values are fine-tuned to achieve such a high-accuracy prediction. These parameters are primarily specific to the data that have been used in an ML method. This effective fine-tuning of the parameters is known as ’learning the model’. Deep learning (DL) [12] is a section of ML. The input feature information is transformed in a hierarchical fashion so that a DL model can learn the representation by organising the lower-level information to a gradually higher-level one. The key idea is that this in-between transformation that bridges the map between input and output prediction should have minimal human feature engineering involvements from the input to the final prediction. It can be composed using deep neural networks, which can use a considerable amount of hyper-parameters to learn the useful features from the input data directly as a complex function of functions. It can be argued that it mimics the human learning process as we also gradually develop our knowledge over the iteration of different fundamental knowledge bases. Because of this, DL is an essential topic in the artificial intelligence domain.

Multimodality can help us deal with the *heterogeneity* aspect of EHRs. Multimodality investigates the data in all the associated modalities or forms. It can help us provide in-depth insights into the diagnosis of a CDSS. It can reveal new insights and discoveries. Multimodality enriches the EHR’s usage capability [13]. Typical medical diagnosis processes are multifaceted; therefore, a multi-modal approach provides a holistic method for diagnosis, resembling a real-world scenario instead of using only one modality [14]. Moreover, recent ML and DL methods are well-adjusted to working with multi-modal data to utilize this useful concept in a prediction model for CDSS [15,16].

The *security and privacy* issue can be handled using the federated learning (FL) concept. FL can be described as different distributed ML approaches where the data are distributed among several individual devices. The training process of these data is specific to the respective devices only. Usually, a central server is used to manage the whole training process, but it is strictly enforced that this server is never allowed to view or obtain the data. The challenging part is effectively aggregating different locally trained models to improve overall performance [17]. FL is practical, operational cost saving, secure as the ownership control can be fully employed, and private as the absolute minimum usage or exposure of the data is allowed [18,19].

Based on the discussion mentioned earlier, we need to investigate the IoMT setup to obtain improved CDSSs with the effective utilization of EHRs using the ML/DL, multimodality, and FL techniques. However, as described in Section 2, it can be observed that the empirical investigation and subsequent evaluation of such setup in the real-time low-end edge computational devices are in scarcity. The usability of the resource-constrained edge computational devices should be investigated more as this can expand the reach of IoMT to a much wider audience (different stakeholders) compared to the current high-end device setup we have. To address this issue, we have attempted to address the following research question in this study:

“What is the performance difference of the low-end edge devices in a federated IoMT-based CDSS application for the early detection of sepsis diagnosis prediction task?”

We choose early detection of sepsis as an example of an IoMT-based CDSS application because of its relevance to this setup mentioned above. We refer to this application as *FedSepsis*. Sepsis can be described as a potentially life-threatening condition. Sepsis is caused by the body’s overwhelming responses to infection. It is one of the major reasons for mortality and morbidity in the hospital [20]. The survival depends almost entirely on the earliest intervention by applying appropriate antimicrobial treatment [21]. The chance of mortality increases by 7.6% when the treatment is delayed for every hour [22], which means it is extremely crucial to detect it as early as possible.

In this study, we use two different kinds of low-end edge devices, namely Raspberry Pi [23] and Jetson Nano [24] to implement *FedSepsis*. We have used multi-modal EHR data and utilized different state of the art DL and natural-language processing (NLP) methods [25] to handle the sparsity and heterogeneity issue. Two different types of federated aggregation techniques are studied to investigate their applicability. Our previous work attempted to address the early sepsis detection in a single-server setting with single-modal data [26]. We also attempted to investigate the performance of the Raspberry Pi device in an FL setting with the basic aggregation technique [27]. *FedSepsis* shows that the results provide a clear superiority to using the multi-modal data over the single-modal data to detect sepsis. It also suggests that using different types of low-end edge devices can provide better performances in an FL setup. We also report the other system-level performance for different edge devices. Overall, our study indicates that IoMT setup using low-end edge computational devices for CDSSs can be a promising new prospect in the future.

The remainder of this article is organized as follows. Section 2 highlights the importance of exploring the usage of low-end edge devices by summarizing the current research on this. Section 3 provides an overview of the different methods and data processing we used for implementing *FedSepsis*. We also provide a detailed description of the sepsis-detection problem here. In Section 4, we discuss the experimental setup of *FedSepsis* application and explain the evaluation metrics used in different experiments. Section 5 reports the results of different experiments. Here, we also report critical system-level information about the Raspberry Pi and Jetson Nano devices. Finally, in Section 6, we discuss the results and their importance in a broader context.

The code implementation of *FedSepsis* can be obtained using the following link (accessed on 6 January 2023): https://github.com/anondo1969/FedSepsis.

## 2. Related Works

The primary focus of our study is to investigate the usability of the low-end edge devices in a federated IoMT-based CDSS application for CDSS tasks. In this section, we summarized the importance of low-end edge devices by analyzing the recent related works.

Kairouz et al. [17] provided an in-depth overview of the advances and open problems in FL. One key challenge identified in the platform development and deployment area is the difference in hardware among different devices. They mainly focused on the performance and device stability and mentioned that running computation must not affect it. Communication, storage, and computational capabilities of different devices are categorized as a core challenge in an FL setting by Li et al. [28]. Their primary concerns were the power (battery level) and hardware (CPU (central processing unit), memory). Scarce computational resources and relatively small storage capacity of the devices are mentioned by Bonawitz et al. [29], discussing the scaling issue of federated learning with the practical system design case. Gao et al. [30] used Raspberry Pi devices and investigated the performance of speech commands and electrocardiogram-related tasks in the FL setup. Orescanin et al. [31] investigated the fine-tuning performance in FL setting using Raspberry Pi devices using a pre-trained MobileNetV2 model on the CelebA dataset. The comparison of different edge devices was not performed in these works. The abovementioned discussions indicate the challenges to using low-end computational devices and highlight why it should be explored more in an FL setting to obtain the best performance.

## 3. Methods and Materials

In this section, we discuss the data and different methods used to conduct the experiments to address the research question described in Section 1 to implement *FedSepsis*.

### 3.1. Dataset

We used EHRs from the MIMIC-III (Multiparameter Intelligent Monitoring in Intensive Care) database, version 1.4 [32]. It comprises over 58,000 hospital admissions of over 45,000 patients between June 2001 and October 2012.

#### 3.1.1. Data Selection Criteria

**Figure 1:**
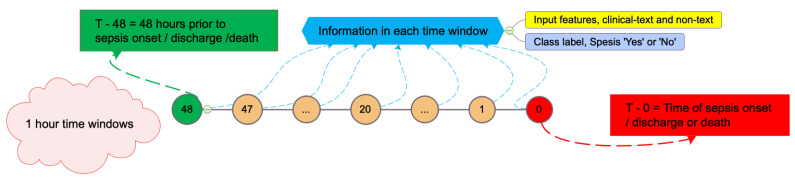
A *care episode* having with 48 *time-window*s (*care episode* length = 48).

**Figure 2:**
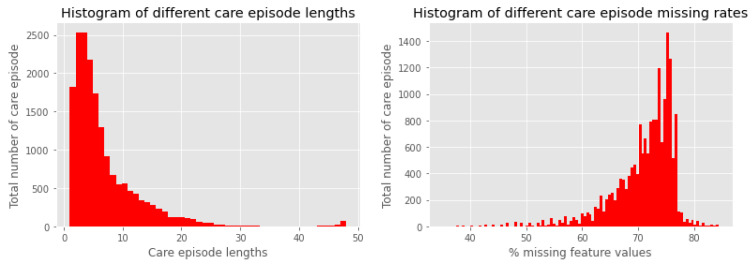
Histogram of different *care episode* lengths (**left**) and missing rates (**right**).

Patients older than 15 years were considered and the data were taken up until the first sepsis onset (see Section 3.1.2 for details), death or being discharged from the hospital. From this end point up until 48 h of continuous data were considered. We denote one such continuous event as a *care episode* where the total time (maximum of 48 h) is binned into one hour intervals. This interval is denoted as the *time-window*. Figure 1 illustrates one *care episode* with 48 one-hour *time-window*s. One should notice that the *care episode* length (total time) varies as can be seen in Figure 2.

#### 3.1.2. Sepsis Definition

We used the Sepsis-3 clinical criteria [20,33] to define sepsis. With these criteria, the sepsis onset time is regarded as the latest *time-window* where both *organ dysfunction* and *suspected infection* criteria are satisfied. *Suspected infection* is fulfilled when at least two doses of antimicrobial treatment are given and any culture taking test is ordered. If the antimicrobial treatment is given first, then the culture test must be conducted within 24 h. On the other hand, if the culture test was conducted first then the antimicrobial treatment must be initiated within 72 h after the culture test. *Organ dysfunction* is measured by the sequential organ failure assessment (SOFA) score [34]. If the SOFA score is greater than or equal to 2 points from its baseline then the condition is denoted as *organ dysfunction*. *Organ dysfunction* is needed to be measured 48 h before to 24 h after the occurrence of the *suspected infection*. The baseline should be considered as the latest value measured before the 72 h *time-window*. If there is no known pre-existing *organ dysfunction* then the baseline should be regarded as zero. As the data are obtained from an intensive care unit (ICU)-based dataset, we can view the sepsis cases as a combination of community-acquired and hospital-acquired cases.

#### 3.1.3. Feature Selection

Both the *clinical-text* and the *non-text* data were considered as features. *Clinical-text* data were collected as all the physician and nursing notes had no error mentioned in the database fields. The *non-text* data consisted of the demographic, vital, and the lab data from the EHRs. Some of the feature values can be extremely sparse as can be seen in Figure 2 (right). Therefore, we only considered the *care episode*s where the features have a less than 90% missingness in the *non-text* data. There is indeed quite a significant amount of missingness to be considered. Because of this, we researched extensively to tackle the missingness to obtain an improved result, as discussed in Section 4.1. Table 1 provides the name of all the features used in the experiments.

### 3.2. Federated Learning

**Figure 3:**
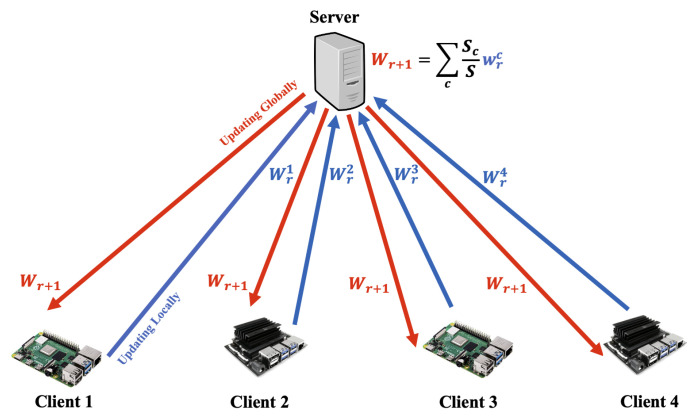
Federated learning architecture.

Figure 3 illustrates the federated learning framework architecture we used. Initially the global model is created with some random weights $W_{r}$ from the *server* and then the *server* sends it to all the *client*s. It is trained and updated locally in the *client* devices. The trained model $W_{r}^{c}$ is then sent back to the *server*. The *server* then aggregates all these models to create one global model $W_{r}+1=\sum_{c}\frac{S_{c}}{S}W_{r}^{c}$. Here, *c* is each *client*, *r* is *round*, meaning the process or one cycle to create one aggregated global model. Usually there are multiple *round*s to find the best trained and aggregated global model. *S* is the total sample size in the dataset. $S_{c}$ is the sample size in each *client*. We have used the following aggregation techniques.

#### 3.2.1. Federated Averaging (Simple)

The aggregation technique is described as the *FederatedAveraging* [35] algorithm as shown in Figure 3. In this algorithm, the different locally trained models are aggregated using the weighted average of all the models.

#### 3.2.2. Federated Optimization (Opt)

The aggregation technique is described as the *FEDOPT* algorithm [36], where the global model is updated in the server by applying the global optimizer (usually a gradient based one) using the average of the local models.

### 3.3. Machine Learning Methods

We used several state-of-the-art machine learning methods for the experiments. Recurrent neural network-based long short-term memory networks (RNN-LSTM) [37,38] were used for the sepsis prediction task. Generative adversarial imputation nets (GAINs) [39] were used for the missing *non-text* data imputation. Two different bidirectional encoder representations [40,41] from transformers (BERTs) [42] models were used to generate *text embedding*s from the *clinical-text* data.

#### 3.3.1. Long Short-Term Memory Networks (RNN-LSTM)

*Care episode* information is time-series data which means it is sequential. To predict sepsis as early as possible from a given *time-window*, we are allowed to use only the information we had in the previous *time-window*s. A suitable DL model for this case can be recurrent neural networks (RNNs) [37] because of their capability of preserving and restoring information in its internal memory. A carefully constructed loop of all the *care episode*s is calculated by iterating all the *time-window*s in RNNs. Therefore, to make a prediction, it can use only the current and all the previous *time-window*s to detect sepsis. We need to be careful about two aspects of RNNs, namely, the exploding gradients problem and the vanishing gradients problem. The first problem occurs when RNNs assign an unusually high value to the model weights. The second problem can happen when this value is unreasonably low, which is relatively common in long sequences or, in our case, *care episode*s with a relatively high length.

Long short-term memory (LSTM) networks [38] provide solutions for these two critical problems. Its memory can be extended to learn essential and relevant information, even if it resides in long past *time-window*(s). Three gated cells (*input*, *forget*, and *output*) are used here to ensure the specific information to store or delete. The *input* gate is responsible for information gaining, the *forget* gate for detecting non-relevant information, and the *output* gate ensures the previous outputs are used as important ones. One could notice that it is similar to the medical diagnosis process as, in most cases, recent information is given more priority to diagnose an outcome.

Equation (1) represents the feedforward networks or multi-layer perceptron (fully connected neural networks). $X_{i}$ is the input.

(1) $$h=f(X_{i})$$

Usually, a hidden state is needed to preserve the previous information from a *care episode*. This state can be interpreted as additional features from the previous *time-window*s. If we want to predict whether the current stage is positive or not at a given *time-window* then we need to consider both the input $X_{t}$ and the hidden state from the previous time step $h_{t-1}$, as can be seen in the following equation:

(2) $$h_{t}=f\left(x_{t},h_{t-1}\right)$$

*h* is the hidden states (1) composed from the previous *time-window*s. We can use it to (2) predict the subsequent outcome. Usually, LSTM sub-divides these two usages into two distinct variables $h_{t}$ and *C*. *C* symbolizes the LSTM hidden cell. Now we can control the information flow using the three gates we mentioned above,

(3) $$gate_{forget}=\sigma \left(W_{fx}X_{t}+W_{fh}h_{t-1}+b_{f}\right)$$

(4) $$gate_{input}=\sigma \left(W_{ix}X_{t}+W_{ih}h_{t-1}+b_{i}\right)$$

(5) $$gate_{out}=\sigma \left(W_{ox}X_{t}+W_{oh}h_{t-1}+b_{o}\right)$$

If we need to update the cell state and hidden state, then we can use the following equations,

(6) $$C^{*}=\tanh\left(W_{cx}X_{t}+W_{ch}h_{t-1}+b_{c}\right)$$

(7) $$C_{t}=gate_{forget}\cdot C_{t-1}+gate_{input}\cdot C^{*}$$

(8) $$h_{t}=gate_{forget}\cdot \tanh\left(C_{t}\right)$$

σ(x) and tanh(x) are known as activation functions. σ(x) or sigmoid function can take any real-valued input and produces output in a range between 0 and 1,

(9) $$\sigma \left(x\right)=\frac{1}{\left(1+exp(-x)\right)}$$

If the output sum of a sigmoid function is equal to one, then it is known as the soft-max function. Usually, a log form of soft-max function is used for prediction for multi-label classification because we can then consider the outputs as probability scores. Therefore it can be regarded as a probabilistic classification. tanh(x) also takes a real-valued input and provides the output in a range between [−1,1],

(10) tanh(x)=2σ(2x)−1

#### 3.3.2. Generative Adversarial Imputation Nets (GAIN)

Generative adversarial imputation nets (GAINs) [39] are used for the imputation of the missing data. GAIN is a generalization of the GAN (generative adversarial networks) [43] architecture. Following the GAN architecture principle, in GAIN the generator attempts to impute the missing data whereas the discriminator tries to minimize the classification loss between the actual and observed imputations. In the GAIN architecture, a discriminator is assisted with additional ‘hints’ to ensure that the samples are being generated according to the real implicit data distribution.

#### 3.3.3. Bidirectional Encoder Representations from Transformers (BERT)

BERT (bidirectional encoder representations from transformers) [42] is a deep learning model based on the transformer–encoder architecture [44] that pre-trains language representation with the use of a huge amount of data. It can be used to generate text features (*text embedding*) from a given text which can be used for a later classification task using different machine learning-based models (downstream tasks). BERT can also be used directly for a number of NLP classification tasks such as next sentence prediction, name–entity recognition, and question-answering. A self-attention mechanism is used in the transformer–encoder architecture where the model’s pre-training objective function is composed of mask language modeling and next sentence prediction unsupervised tasks. Stochastic optimization is used to estimate the *text embedding*s and model parameters. Further fine-tuning can be required to perform problem-specific downstream tasks.

We have used two such fine-tuned BERT modes for generating the *text embedding*s from *clinical-text* data. These *text embedding*s are later used to predict the early detection of sepsis. In this paper, we refer to these models as *ClinicalBERT-Alsentzer* [41] and *ClinicalBERT-Huang* [40]. Both *ClinicalBERT-Alsentzer* and *ClinicalBERT-Huang* used the clinical texts from the MIMIC-III v1.4 database [32].

*ClinicalBERT-Alsentzer* aggregates the 15 types of clinical texts into discharge summary type or non-discharge summary type data and then tokenized them using the *ScispaCy* [45]. These tokenized sentences were used to fine-tune the original BERT base model. *ClinicalBERT-Huang* additionally fine-tuned the model for the hospital-readmission prediction task. In BERT, the maximum token length is 512 which can be limited for long clinical texts. *ClinicalBERT-Huang* tackles it by splitting the long text and combining these multiple predictions for one global prediction in an efficient way.

## 4. Experimental Setup

In this section, we discuss several aspects of the experimental setup to address the research question described in Section 1.

### 4.1. Missingness Representation in the Care Episode

A *time-window* in a *care episode* (Figure 1) can contain multiple input-feature values or no values at all. We need to represent this missingness in an efficient way for a better prediction as our data are quite sparse (Figure 2). We have devised two methods for this missing-data imputation task based on the randomness notion. We used two strategies for the missing not-at-random case. One is using GAIN (discussed in Section 3.3.2) to generate the imputation data (*GAIN-imputation*). For the other case we did not impute but used an integer value which is entirely absent in the whole dataset (*distinct-value*). The goal is that this integer should indicate that the value is missing for a particular reason or missing not-at-random. For the missing at-random case we performed the carry-forward of the present data to all the next windows as long as we do not see another value in the forthcoming window. The later value is then again carry-forwarded if any later *time-window* value is missing. If the missing value for a particular input feature is presented in the whole *care episode* then we use the global average of that particular value across the whole data (*carry-forward-mean-imputation*).

### 4.2. Training–Testing–Tuning Data Generation

The total data are subdivided into three portions for the training (80%), testing (10%), and tuning (10%) of the prediction model. We kept both the sepsis class-based and *care episode* length-based distribution similar in these three datasets. Positive class is considered when the Sepsis-3 criteria is met. Because of this distribution similarity ensurity, we omitted 50 *care episode*s where a particular *care episode* length contains five or fewer cases. Table 2 provides the overview of the data. We can see that the negative episodes are almost 90% which provides us with highly-imbalanced datasets and thus makes the sepsis (positive class) prediction quite challenging.

### 4.3. Text Embeddings Generation from Clinical Texts

We used both *ClinicalBERT-Alsentzer* and *ClinicalBERT-Huang* (Section 3.3.3) to generate the *text embedding*s from the clinical text. All the *clinical-text*s in each *care episode* were tokenized first (up to 512 characters for each text). We then used it for the inference in the ClinicalBERT models. The values from the last four hidden layers of the ClinicalBERT(s) were considered to generate the feature vectors. Two approaches were taken to create the final *text embedding*s. First, the feature vector was generated from the average of all the layers (with a total length of (768). This is referred to as *short-text-embedding*. Second, the feature vector was generated from the concatenation of all the layers (with a total length of 768 ∗ 4 = 3072). This is referred to as *long-text-embedding*.

### 4.4. RNN-LSTM and GAIN Hyper-Parameter Tuning

We tuned the hyper-parameters for RNN-LSTM and GAIN models as described in Table 3. We randomly selected 100 data points among these parameters and thus conducted 100 experiments with the training data to find the best tuning parameter values. We oversampled the distribution in the training data to make the data balanced (50% positive) in each mini-batch. We used tuning data for evaluation and used AUPRC (area under the precision recall curve, described in Section 4.7) to select the best-tuned model. Based on these fine-tuned parameters we finally evaluated the trained model using the testing data.

### 4.5. Configuration of *Server* and *Client* Devices

**Figure 4:**
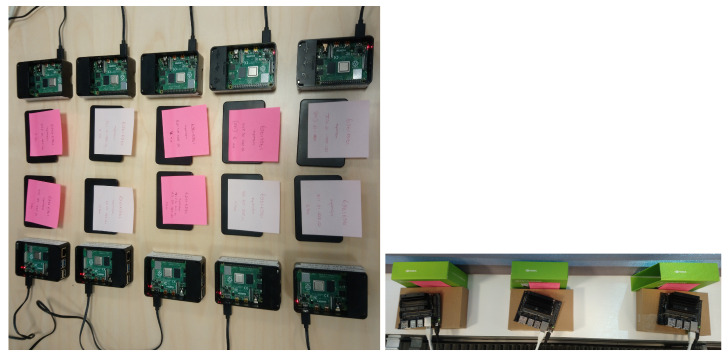
Raspberry Pi (**left**) and Jetson Nano (**right**) edge devices used in the experiments.

For the *Experiment A* (described in Section 4.6.1), which is performed in a single machine we used an Ubuntu 20.04.3 LTS (GNU/Linux 5.13.0-28-generic x86_64) based server with GeForce GTX 1080 Ti graphics processing unit (GPU). For the *Experiment B* (described in Section 4.6.2), an Ubuntu 18.04.5 LTS (GNU/Linux 4.15.0-142-generic x86_64) based server with GeForce RTX 2080 Ti graphics processing unit (GPU) was used as the *server* for the federated learning experiments (*Experiment B*, described in Section 4.6.2). For *client*s, we used two different kinds of edge devices. 10 Raspberry Pi [23] 4b devices (Ubuntu 20.10 as GNU/Linux 5.8.0-1024-raspi aarch64) and three Jetson Nano [24] Developer Kit devices (Linux-4.9.253-tegra-aarch64-with-Ubuntu-18.04-bionic) were used for different experiments. Figure 4 illustrates the *client* edge devices used in different experiments.

Socket programming [46] was used to establish the federated learning (discussed in Section 3.2) setup. In socket programming we can connect two nodes on a network to establish a communication channel. A server usually creates the listener socket where multiple clients can connect. PyTorch [47] framework was used to develop the deep learning models.

### 4.6. Experiments

We conducted several experiments to evaluate the performance of early detection of sepsis using multi-modal data. We compared the performance in a federated learning based setting using two different kinds of edge devices. The experiments are classified into following categories.

#### 4.6.1. Experiment A: Single-Server Setting

**Figure 5:**
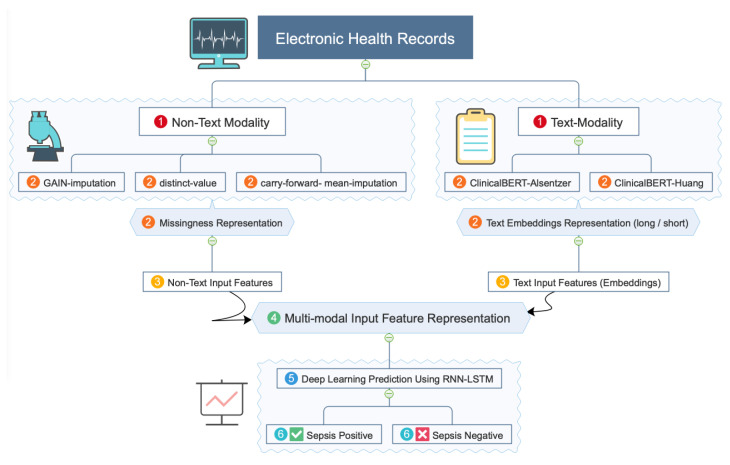
Schematic diagram of the proposed model for the single-server setting. For details please see Section 4.6.1.

We generated the *text embedding*s using the process described in Section 4.3. The *non-text* input features (showed in Table 1) were processed in terms of missingness, as described in Section 4.1. If there were multiple values for a particular feature in a time window, then we used the average value. These clinical features were then used as the input for the RNN-LSTM model (discussed in Section 3.3.1). The hyper-parameter tuning process of the deep learning models is discussed in Section 4.4. The RNN-LSTM model predicts the output as a probability score based on the current and previous features presented in a current time window. The threshold of >0.5 was used to determine the sepsis or non-sepsis case for one *time-window* and once it obtains a positive prediction then the later time window prediction scores are ignored. Three different RNN-LSTM models were considered based on the difference of the input features, namely *multi-modal*, *non-text*, and *clinical-text*. We concatenated the *text embedding*s and the *non-text* processed features for the multi-modal representation of the input features. The mini-batch size used for training *non-text*, and *clinical-text* models was 1000, and for *multi-modal* model it was 500. Figure 5 illustrates the schematic diagram of the proposed model for the single-server setting.

#### 4.6.2. Experiment B: Federated Learning Setting

The training data were split equally among all the *client* devices. We then performed *Experiment A* (Section 4.6.1) in a federated learning setting. Due to the computational limitation constrain compared to the server, the mini-batch size used for training *multi-modal* and *clinical-text* models was 500, and for *non-text* model it was 1000. The details about the devices and federated learning setting is discussed in Section 4.5. Both *simple* and *opt* (described in Section 3.2) aggregation techniques were used to create the global RNN-LSTM model. Due to time-constraint issue we only conducted the experiments compared to the best three models (based on AUPRC score) obtained from *Experiment A* (Section 4.6.1). As the training dataset is relatively small, therefore to overcome the overfitting issue we used a total of five *round*s for each experiment. Along with the evaluation results we also report the system information of the *client* devices.

### 4.7. Evaluation Metrics

There are four possible outcomes for binary classification, with a positive and a negative class. If the model assigns the positive class to a positive example then it is a true positive (TP). If the model assigns the positive class to a negative example then it is a false positive (FP). If the model assigns the negative class to a negative example then it is a true negative (TN). If the model assigns the negative class to a positive example then it is a false negative (FN).

Now we can calculate the precision and recall in the following ways,

(11) $$Precision=\frac{TP}{TP+FP}$$

(12) $$Recall=\frac{TP}{TP+FN}$$

Area under the receiver operating characteristic curve (AUROC) and area under the precision–recall curve (AUPRC) were used to evaluate the model performance.

For a classification task with a positive and a negative class, a model will estimate the probability for an example belonging to the positive class, and if this value surpasses a threshold, the example will be classified as positive. The AUROC score is based on the true positive rate (the proportion of correct classifications of positive examples) and the false positive rate (the proportion of negative examples classified as belonging to the positive class). By varying the threshold for when an example is considered as belonging to the positive class, pairs of true positive rates and false positives rate will be obtained. These pairs can be plotted as a curve, with the false positive rate on the x-axis, and the true positive rate on they y-axis. This curve is known as the receiver operating characteristic and the AUROC value corresponds to the area under this curve.

Reviewing both precision and recall is useful in cases where there is an imbalance in the observations between the two classes. Specifically, there are many examples of no event (class 0) and only a few examples of an event (class 1). The reason for this is that typically the large number of class 0 examples means we are less interested in the skill of the model at predicting class 0 correctly, e.g., high true negatives. The key to the calculation of precision and recall is that the calculations do not make use of the true negatives. It is only concerned with the correct prediction of the minority class, class 1. A precision–recall curve is a plot of the precision (y-axis) and the recall (x-axis) for different thresholds.

The *earliness* in predicting sepsis is calculated based on the median prediction time from the sepsis onset in hours for all the true positive cases. A prediction score threshold of >0.5 is used to determine the correct prediction, and we retained the first positive *time-window* value of the *care episode* to calculate *earliness*. Therefore, the larger value of *earliness* represents better prediction in general. As *earliness* is calculated based on true positives only, we should consider it together with AUROC and AUPRC values to obtain a more balanced overview of the model’s performance.

Table 4 shows the *client* device system information topics that we reported in Section 5.3.

## 5. Results

In this section, we present the results obtained by conducting different experiments discussed in Section 4.6.

### 5.1. Experiment A Results: Single-Server Setting

Table 5 shows the results obtained using only the *non-text* data. We can see that *GAIN-imputation*-based missingness representation provides the best result and *carry-forward-mean-imputation* provides the worst result. The best evaluation scores obtained as AUPRC: 87.30%, AUROC: 98.31%, and *earliness*: 4.45 h.

Table 6 shows the results obtained using only the *clinical-text* data. We can see that the result is comparatively worse and it is quite challenging to select a better model as different evaluation metrics provides best results in different *round*s. The best evaluation scores obtained as AUPRC: 14.06%, AUROC: 59.99%, and *earliness*: 7.28 h.

Table 7 shows the results obtained using both the *clinical-text* and *non-text* data. It can be seen that using multi-modal setting provides the best results. The best evaluation scores obtained as AUPRC: 96.55%, AUROC: 99.35%, and *earliness*: 4.58 h.

### 5.2. Experiment B Results: Federated Learning Setting

We have used two different types of edge device to conduct the experiments in federated learning setting. The results are reported here.

#### 5.2.1. Raspberry Pi

Table 8 shows the results obtained using *non-text* data in a federated learning setting using 10 Raspberry Pi devices. We can see that federated aggregation technique *simple* performed better over *opt*. The best evaluation scores obtained as AUPRC: 71.39%, AUROC: 97.60%, and *earliness*: 4.54 h.

Table 9 shows the results obtained using *clinical-text* data in a federated learning setting using 10 Raspberry Pi devices. Although the result is comparatively worse, but similar to before the result improves after each *round* and aggregation technique *simple* performed better over *opt*. The best evaluation scores obtained as AUPRC: 14.62%, AUROC: 58.91%, and *earliness*: 7.73 h.

Table 10 shows the results obtained using *multi-modal* data in a federated learning setting using 10 Raspberry Pi devices. *multi-modal* model provides the best results. It is worth noting that the best AUPRC score was obtained after the *round* 4. The best evaluation scores obtained as AUPRC: 84.80%, AUROC: 98.49%, and *earliness*: 4.54 h.

**Figure 6:**
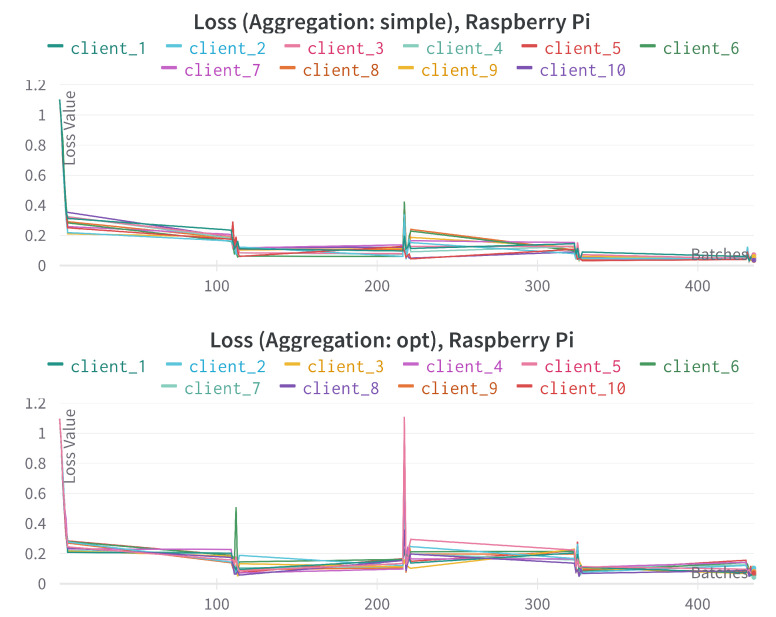
Loss comparison for different federated aggregation techniques using 10 Raspberry Pi devices. The x-axis represents the incremental number of training mini-batches, the y-axis represents loss-values.

Figure 6 provides the loss comparison for different federated aggregation techniques using 10 Raspberry Pi devices for the training of the *multi-modal* model. We can see that the loss value gradually decreased after each *round* and it is much smoother in the case of the aggregation technique *simple* over *opt*.

Table 11 shows the results obtained using *multi-modal* data in a federated learning setting using different number of Raspberry Pi devices. It provides insights into the empirical settings regarding data and the number of devices. We can see that the best results were obtained using only one or two devices, and after increasing it to six and more devices the AUPRC score decreased. This suggests that using a relatively small amount of data can increase the chance of overfitting, and we have to be careful in that case. Choosing less number of devices may be a walk around in that case. The best evaluation scores obtained using two devices as AUPRC: 99.20%, and AUROC: 99.91%, and using eight devices the *earliness* score is 4.53 h.

#### 5.2.2. Jetson Nano

Table 12 shows the results obtained using *non-text* data in a federated learning setting using three Jetson Nano devices. Similar to the Raspberry Pi case, federated Aggregation Technique *simple* provided the best results. We can also see that fewer devices provide better results than using more. The best evaluation scores obtained as AUPRC: 94.43%, AUROC: 99.51%, and *earliness*: 4.53 h.

Table 13 shows the results obtained using *clinical-text* data in a federated learning setting using three Jetson Nano devices. We can observe that the performance is significantly worse, similar to the Raspberry Pi cases. It suggests that it may be possible that the text modality alone sometimes may not be a wise choice as input features. The best evaluation scores obtained as AUPRC: 15.12%, AUROC: 63.58%, and *earliness*: 7.73 h.

Table 14 shows the results obtained using *multi-modal* data in a federated learning setting using three Jetson Nano devices. Once again, the multi-modal representation of the input features provided the best results. Compared to Table 11 the results with three devices provides a better AUPRC score. The best evaluation scores obtained as AUPRC: 98.99%, AUROC: 99.89%, and *earliness*: 4.56 h.

**Figure 7:**
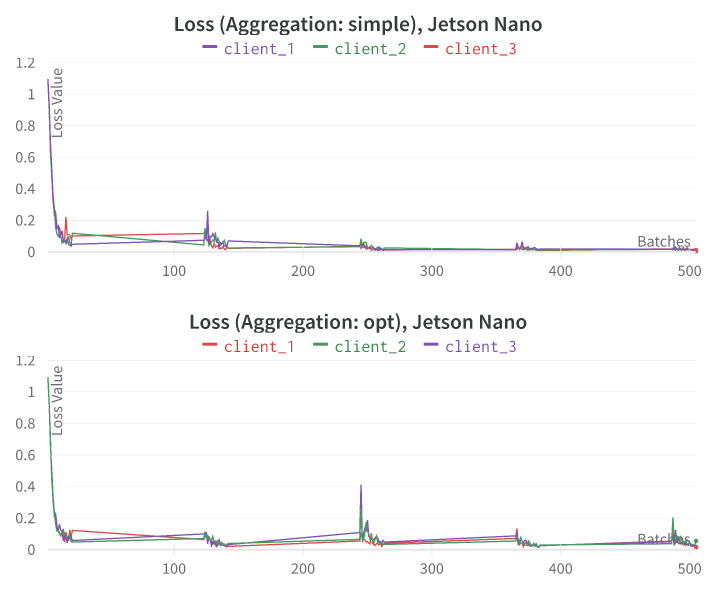
Loss comparison for different federated aggregation methods using Jetson Nano devices. x-axis represents the incremental number of training mini-batches, y-axis represents loss-values.

Figure 7 provides the loss comparison for different federated aggregation techniques using three Jetson Nano devices for the training of *multi-modal* model. We can see that the loss value was gradually decreased after each *round* and it is much smoother in the case of the aggregation technique *simple* over *opt*.

Table 15 shows the results obtained using *multi-modal* data in a federated learning setting using different Jetson Nano devices. As it can be observed, with two devices, the best AUPRC score was obtained. The best evaluation scores obtained as AUPRC: 99.36%, AUROC: 99.93% with two devices, and *earliness*: 4.51 h with three devices. The best performance here is slightly worse than the best performance obtained with the similar experiments using Raspberry Pi devices, as can be compared with Table 11.

### 5.3. Edge Device System Information

In this section, we report different edge device system information as mentioned in Table 4. For the Raspberry Pi, the experiment was conducted with 10 devices (see Table 10), and for the Jetson Nano, three devices (see Table 14) were used. For both cases, *multi-modal* input features and *simple* federated aggregation method were used.

**Figure 8:**
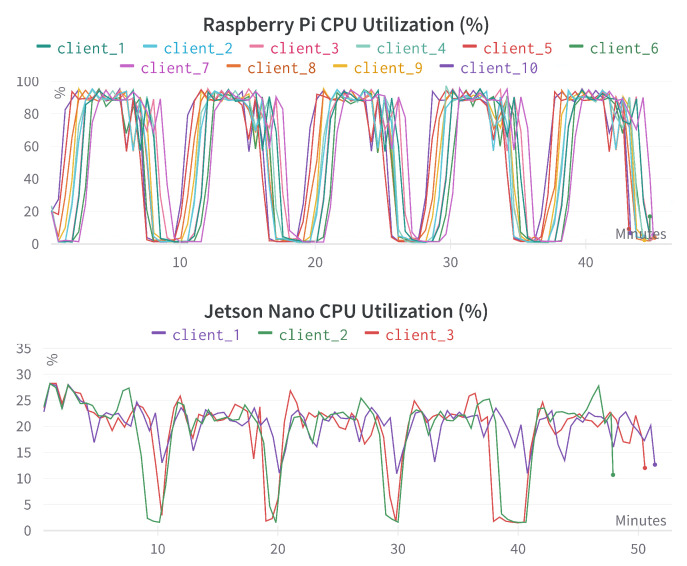
CPU utilization comparison for different Raspberry Pi and Jetson Nano devices. The x-axis represents the time in minutes, the y-axis represents the CPU utilization percentage (%).

Figure 8 provides the CPU utilization comparison for different Raspberry Pi and Jetson Nano devices. We can see the precise segmentation of each *round* (of a total of five). Raspberry Pi’s CPU utilization is much higher, and it is consistent across all 10 devices. This is understandable as Jetson Nano uses GPU. Therefore, the majority of computational works are performed there. This indicates that we need to be careful if we plan to run other resource-heavy computations on both devices, mainly Raspberry Pi.

**Figure 9:**
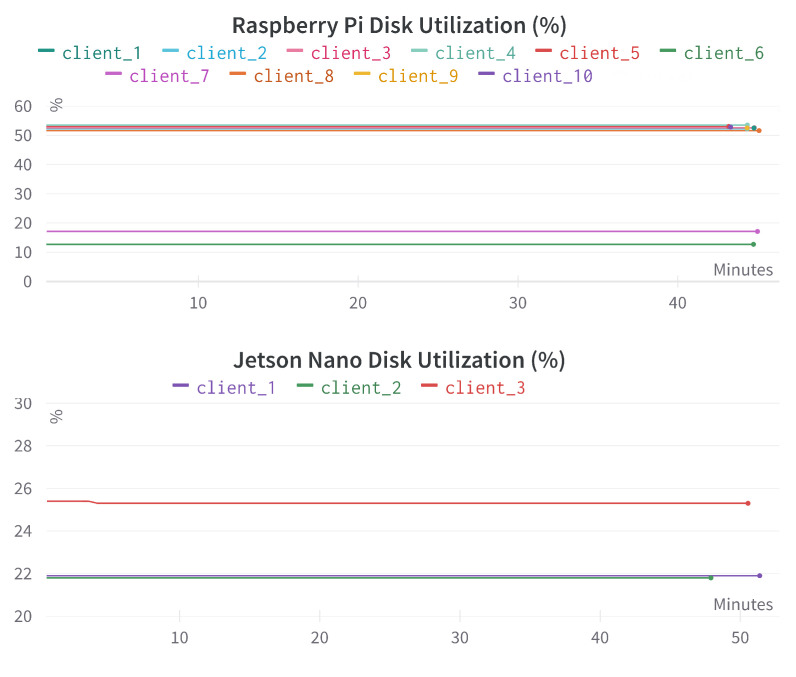
Disk utilization comparison for different Raspberry Pi and Jetson Nano devices. The x-axis represents the time in minutes, the y-axis represents the disk utilization percentage (%).

Figure 9 provides the disk utilization comparison for different Raspberry Pi and Jetson Nano devices. The total storage memory capacity for 8 of the 10 Raspberry Pi devices was 40 Gigabytes (GB). The rest had a capacity of 128 GB. All three Jetson Nano devices had a storage memory capacity of 128 GB. We can see that the disk utilization is also consistent in all the relatively low-storage memory devices. Even with a 40 GB total capacity, the disk utilization is below 60% for the Raspberry Pi devices. This suggests that we need to optimize the disk storage given the dataset size.

**Figure 10:**
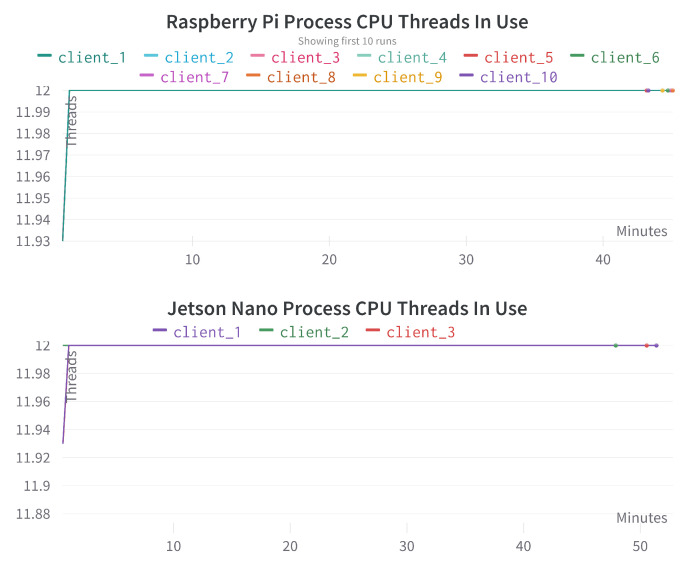
Process CPU threads in use comparison for different Raspberry Pi and Jetson Nano devices. The x-axis represents the time in minutes, the y-axis represents the number of CPU threads in use.

Figure 10 provides the comparison regarding the number of ’CPU process threads in use’ for different Raspberry Pi and Jetson Nano devices. We can see that it is entirely consistent across all the devices, and in almost all the cases, it is close to 12. It suggests that if we can effectively utilize all the cores of the CPU, then we may obtain a faster and better parallel performance, as can be seen in these graphs.

**Figure 11:**
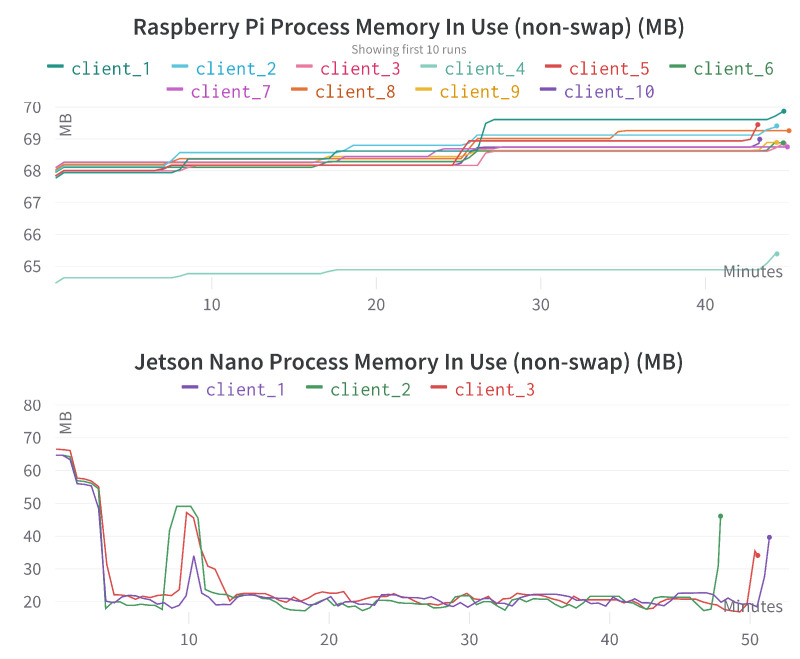
Process memory in use comparison for different Raspberry Pi and Jetson Nano devices. The x-axis represents the time in minutes, the y-axis represents the used process memory in megabytes (MB).

Figure 11 provides the comparison of process memory in use for different Raspberry Pi and Jetson Nano devices. Process memory is the collection of processes responsible for obtaining, preserving, and retrieving information. We can see that process memory utilization is relatively low in Raspberry Pi devices, and it is even much less (with a bit of irregularity) in Jetson Nano devices.

**Figure 12:**
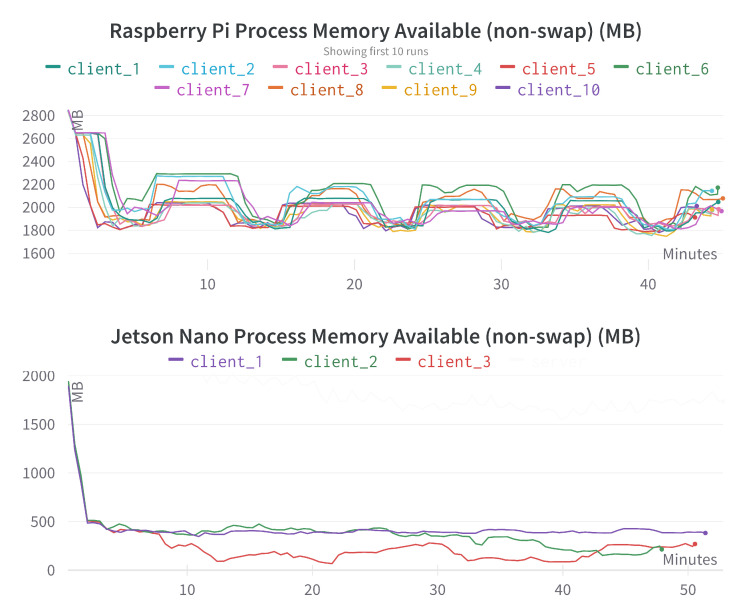
Process memory available comparison for different Raspberry Pi and Jetson Nano devices. The x-axis represents the time in minutes, the y-axis represents the available process memory in megabytes (MB).

Figure 12 provides the process memory available comparison for different Raspberry Pi and Jetson Nano devices. If we observe this figure jointly with Figure 11 then we can see that Raspberry Pi uses significantly less process memory compared to Jetson Nano devices. Even with the small dataset and relatively small DL models used in *FedSepsis* experiments, it takes almost all the process memory in Jetson Nano. Therefore, Raspberry Pi is suited for more data and more complex training models.

**Figure 13:**
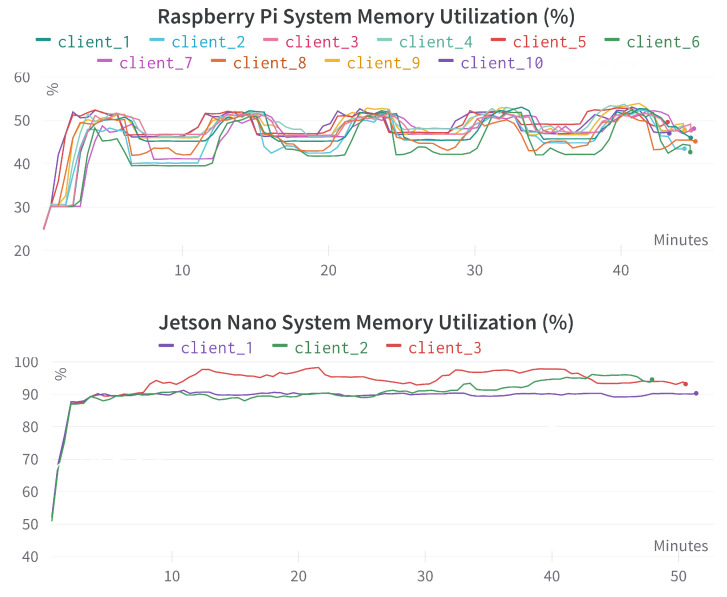
System memory utilization comparison for different Raspberry Pi and Jetson Nano devices. The x-axis represents the time in minutes, the y-axis represents the system memory utilization in percentage (%).

Figure 13 provides the system memory utilization comparison for different Raspberry Pi and Jetson Nano devices. System memory or random access memory (RAM) stores data and instructions temporarily so that the CPU can use it later by accessing it randomly. Similar to process memory, system memory utilization is also much higher in Jetson Nano devices than Raspberry Pi devices. We need to be very cautious about the dataset size (mini-batch size) and the complexity of the DL training model while utilizing Jetson Nano devices.

**Figure 14:**
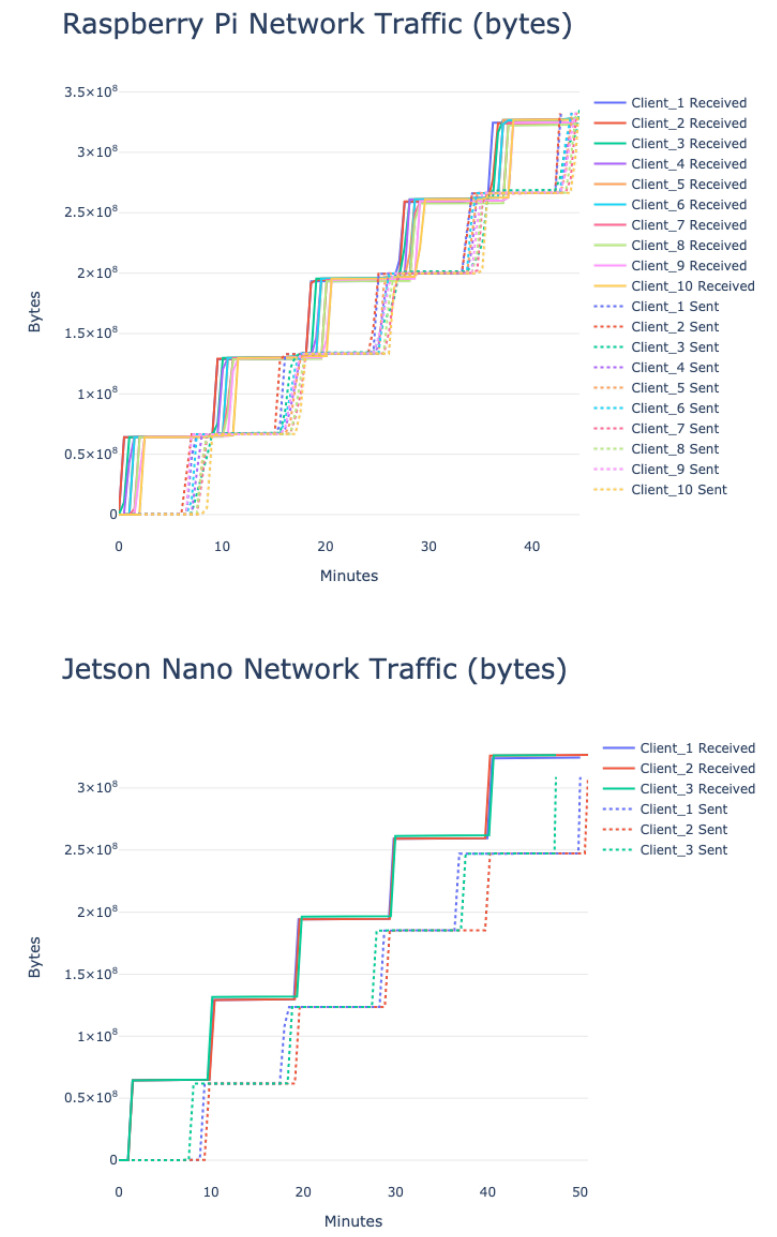
Network traffic comparison for different Raspberry Pi and Jetson Nano devices. The x-axis represents the time in minutes, the y-axis represents network traffic in bytes.

Figure 14 provides the network traffic comparison for different Raspberry Pi and Jetson Nano devices. The data-size pattern is very similar as we exchanged the same types of information for every *round*. If the model is more complex, the data size will be bigger. Therefore, network usage should be carefully constructed and monitored.

**Figure 15:**
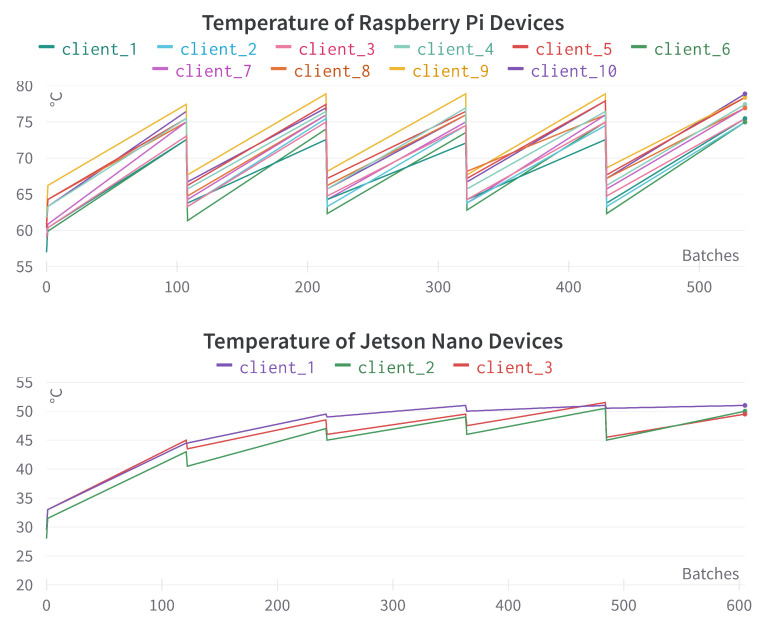
Temperature comparison for different Raspberry Pi and Jetson Nano devices. The x-axis represents the incremental number of training mini-batches, the y-axis represents temperature in Celsius (${}^{\circ }$C).

Figure 15 provides the temperature comparison for different Raspberry Pi and Jetson Nano devices. We can see that the temperature obtains significantly high for Raspberry Pi devices. As we used a relatively small dataset and simple DL training model there, on average, it took 40 to 50 min to complete one *multi-modal* training. Therefore, we could provide sufficient time-gap to cool down the devices. In a more practical scenario, using a cooling fan is advisable due to this high temperature during the training process.

## 6. Discussion

In this study, we introduced *FedSepsis*, an FL-based IoMT application for the early prediction of sepsis using DL and EHRs. We utilized two recent federated aggregation algorithms for the FL setup. We incorporated several cutting-edge concepts and methods, such as multimodality, ClinicalBERT for the NLP, RNN-LSTM neural networks, and GAN to implement *FedSepsis*. Moreover, to investigate the aspects of practical usability, we deployed two popular low-end edge devices, namely Raspberry Pi and Jetson Nano. To provide a more balanced and multifaceted evaluation, we presented the results using AUPRC, AUROC, and *earliness* in terms of hours. We also reported system-level information on the low-end devices to provide insights into using such devices in a practical setting. Instead of focusing on the model improvement only, we also emphasized the applicability aspect by integrating cutting-edge techniques with a keen interest in low-edge devices. Therefore, we heavily invested in analyzing the system-level aspects of these low-edge devices. We expect that by taking this combined approach, our *FedSepsis* application will be more effective and ensure far-reaching benefits.

We used genuine (de-identified) EHRs to extract the input features. EHRs are relatively sparse, or the missingness in EHR is generally quite high, which was in our case also. To tackle this issue, we used a GAN-based technique which proved to be successful as the best results were obtained based on this technique to handle missingness.

Representing the clinical text from the EHRs for prediction tasks is one of the most challenging problems in the NLP domain. To investigate this issue in-depth, we used two latest ClinicalBERT models and two different representations from each. The comparison in terms of best performance is inconclusive as both models performed relatively well along with different representations. This study concatenated the text representations (*text embedding*s) with the *non-text* input features as a combined *multi-modal* input feature. In the future, we will explore different training combinations to attach it to different layers of RNN-LSTM.

Incorporating multimodality provided a precise and superior performance over any form of single-modal input features. It also supports the real-time diagnosis procedure and suggests that in future, we should investigate further in utilizing this concept more efficiently. Effective utilization of the image data is one possible option here. The overall optimization in the training model should also be fine-tuned according to different modalities, as we can see that different modalities may carry different degrees of importance in terms of the overall prediction.

Compared to the FL setting we used, the single-server setting’s storage (one terabyte) and computational capacity were considerably superior to the *FedSepsis* application performance. Therefore, a bit of degrading performance in the FL setup should be taken for granted compared to the single-server setup. We also showed that the performance is close to the single-server setup for this task with a smaller number of devices (five, for example). It suggests that we need to be careful about the device choice and the data distribution in an FL setting not to overfit the model. The model weight aggregation techniques also need to be explored further so that the effective loss optimization can be fine-tuned according to the model or the data we have for a particular task.

For the early sepsis prediction task, we reported AUROC, AUPRC, and *earliness* prediction in hours. AUROC and AUPRC provide a more balanced and clinically relevant evaluation of the prediction as we should be more careful about the true positives. Overall the AUPRC score is satisfactory. However, it should be considered together with *earliness* to understand the effectiveness of the prediction. On average, the *earliness* is close to 4.5 h which is satisfactory given the sparsity of the data and the variance in the *care episode* lengths.

The key aim of this study is to evaluate the performance difference of the low-end edge devices as the research question mentioned in Section 1. We can categorize this performance into model-prediction-level and system-level perspectives.

There is a clear performance difference in model prediction while incorporating GAIN for handling missingness. On average, the AUPRC improved 23.17%, and the AUROC improved 6.89% compared to the next imputation strategy across all *non-text*, *clinical-text*, and *multi-modal* cases in the single-server setting. The combination of *clinical-text* with *non-text* or the *multi-modal* case always provided superior performance in all three settings (single-server, Raspberry Pi, and Jetson Nano). In the single-server setting, for the *multi-modal* case, the AUPRC improvement is 9.25%, and the AUROC improvement is 1.04%, compared to the *non-text* modality. Using Raspberry Pi, the AUPRC improvement is 11.06%, and the AUROC improvement is 0.89%. Using Jetson Nano the AUPRC improvement is 4.56%, and the AUROC improvement is 0.39%. The federated aggregation technique (discussed in Section 3.2) *simple* performed slightly better than the technique *opt*. For the *multi-modal* case, compared to *opt* technique, using Raspberry Pi the AUPRC improvement is 1.86%, and the AUROC improvement is 0.43% in using the *simple* technique. Using Jetson Nano, the AUPRC improvement is 3.99%, and the AUROC improvement is 0.54%. Overall, Jetson Nano provided better performance than Raspberry Pi. For the *multi-modal* case, compared to Raspberry Pi, using Jetson Nano, the AUPRC improvement is 16.55%, and the AUROC improvement is 1.40%. Using fewer devices (three) compared to Raspberry Pi (10) and the GPU could be one reason for this improvement. It is worth noting that different combinations of *ClinicalBERT*-based *text embedding* representation (discussed in Section 4.3) did not provide significant performance differences. It should be investigated further in the future.

Section 5.3 provides an in-depth overview of the performance difference from a system-level perspective. For a faster implementation, Jetson Nano can be a viable choice; on the other hand, Raspberry Pi is less memory exhaustive, given that the temperature issue is effectively handled. Effective CPU utilization can also be achieved by exploring the feature importance issue instead of using all the features. Reducing unimportant features could enhance the speed and efficient CPU utilization. The device system information provides valuable insights regarding the practical usability of *FedSepsis*. CPU utilization, process memory, and system memory are crucial to ensure optimum performance in these low-computational devices, and, therefore, the training model or the data to create input features should be fine-tuned accordingly. Device temperature is also a critical issue to consider to ensure that it runs effectively. Network traffic could be vital, and effective allocation needs to be ensured, especially when we have low power and lossy network (LLN), typical for such IoMT infrastructures.

EHRs are either stored or generated in different medical equipment and servers across different hospital physical locations. We should be very cautious about combining or storing these precious and copious amounts of data in a single place from cost–benefit and security–privacy aspects. As CDSSs can be built with these EHRs, which can substantially positively impact the healthcare system, effective interconnection among the medical equipment and servers should be paramount. IoMT can be one excellent solution for this distributed platform. It can be expanded further with a minimal additional interconnection cost to connect it with other hospitals, even across different countries, while ensuring the utmost security and privacy to create a global impact. This has the potential to revolutionize the healthcare industry with a long-lasting impact to help every stakeholder.

We must be cautious about the security and privacy issue as the EHRs are extraordinarily vulnerable, and breaching the information could bring disastrous after-effects. Therefore, the IoMT platform and applications should ensure the reliability, security, and safety aspects as much as possible [48]. Advanced level data intelligence, supported by cutting-edge DL and ML techniques, should be deployed to enable the IoMT platform and applications to be interactive, stateful, iterative, adaptive, and contextual. Along with NLP, we should also focus on other cross-disciplinary domains to make it more effective, such as speech recognition [49], computer vision [50], dialogue and narrative generation, and human–computer interaction.

Effective data aggregation with the help of metadata aggregation [51], and medical sensors [52] can provide us with new insights. As the inter-relations of different medical conditions or diseases are ubiquitous phenomena among patients (comorbidity), incorporating this aspect (for example, general infection detection from various input sources and outcomes) could be an exciting option to explore further. The optimum use of the low-end edge devices will ensure that these new generation computer systems are cost-effective, patient-centric, robust, and are here to create a long-lasting impact, which will help all parties involved, including the patients and the healthcare professionals.

One potential limitation regarding the acceptance of *FedSepsis* as a CDSS to the medical community is the lack of interpretability of the black box model. The medical significance should be investigated [53] as well while predicting the model to make it more acceptable. We can denote the medical significance of a symptom or disease process as a type of clinical manifestation affecting the regular functionalities of organ systems, tissues, living cells, or organs, for example, in the chest area [54]. We will explore this issue in the future.

## 7. Conclusions

In this study, we presented an Internet of Medical Things-based application, namely *FedSepsis*, for early detection of sepsis as an example of a promising clinical decision support system. We used cutting-edge deep learning methods and relevant concepts, such as multimodality, natural-language processing and federated learning to make a robust and practical application. Moreover, we utilized low-end edge devices, such as Raspberry Pi and Jetson Nano, to show the usability aspects of such applications. Multimodality incorporating *clinical-text* data provided improved performance. Jetson Nano yielded better results from both the prediction-level and system-level perspectives. The results suggest that *FedSepsis* is viable to use and can be an ideal choice given that the interpretability aspect is also ensured to a degree as the prediction scores are entirely satisfactory and the possibility of relatively low-cost device usage can benefit all the stakeholders. In the future, sensor integration, interpreting the medical significance of the prediction, and enhancing the security aspects will be explored.

## Figures and Tables

**Table 1:** List of the data features.

Non-Text			Clinical Text
Vital Data	Demographic Data	Laboratory Data	
Systolic Blood Pressure	Gender	Albumin	Nursing Notes
Diastolic Blood Pressure	Admission Age	Bands (Immature Neutrophils)	Physician Notes
Mean Blood Pressure	Ethnicity	Bicarbonate	
Respiratory Rate	Admission Type	Bilirubin	
Heart Rate	Admission Location	Creatinine	
SpO_2_ (Pulsoxymetry)		Chloride	
Temperature Celsius		Sodium	
Cardiac Output		Potassium	
Tidal Volume Set		Lactate	
Tidal Volume Observed		Hematocrit	
Tidal Volume Spontaneous		Hemoglobin	
Peak Inspiratory Pressure		Platelet Count	
Total Peep Level		Partial Thromboplastin Time	
O_2_ flow		INR (Standardized Quick)	
FiO_2_ (Fraction of Inspired Oxygen)		Blood Urea Nitrogen	
		White Blood Cells	
		Creatine Kinase	
		Creatine Kinase MB	
		Fibrinogen	
		Lactate Dehydrogenase	
		Magnesium	
		Calcium (free)	
		pO_2_ Bloodgas	
		pH Bloodgas	
		pCO_2_ Bloodgas	
		SO_2_ Bloodgas	
		Glucose	
		Troponin T	
		Prothrombin Time (Quick)	

**Table 2:** Dataset distribution.

Data	Positive	Negative	% Positive	% Negative
Training	1352	12,982	9.43	90.57
Testing	181	1643	9.92	90.08
Tuning	172	1624	9.58	90.42
**Total**	1705	16,249	9.50	90.50

**Table 3:** Neural Network Parameters.

Name	Values
hidden layers	1, 2, 3
neurons	512, 800, 1024, 2048
drop out	0, 10, 20, 30
epochs	1, 2
classification function	log-softmax
optimizer	Adam optimizer
GAIN alpha	0.1, 1, 10, 100
GAIN mini-batch	512
GAIN hint rate	0.9
GAIN total iterations	15,000

**Table 4:** Topics of *client* device system information.

Topic	Measurement Unit
CPU Utilization	%
Disk Utilization	%
Process CPU Threads In Use	Number of threads
Process Memory In Use (non-swap)	Megabytes
Process Memory Available (non-swap)	Megabytes
System Memory Utilization	%
Temperature	Celsius
Network Traffic	Bytes

**Table 5:** Evaluation results using *non-text* input features in single-server setting.

Missingness Representation	AUPRC (%)	AUROC (%)	Earliness (h)
GAIN-imputation	87.30	98.31	4.45
distinct-value	18.35	75.62	4.01
carry-forward-mean-imputation	14.22	66.47	4.15

**Table 6:** Evaluation results using *clinical-text* input features in single-server setting.

clinicalBERT	Text Embeddings Representation	Missingness Representation	AUPRC (%)	AUROC (%)	Earliness (h)
Huang	short	GAIN-imputation	14.06	57.51	6.35
Huang	long	distinct-value	13.97	58.50	6.53
Alsentzer	short	carry-forward-mean-imputation	13.70	59.48	7.15
Alsentzer	short	distinct-value	13.46	59.99	6.49
Alsentzer	long	carry-forward-mean-imputation	13.44	56.31	5.77
Alsentzer	long	distinct-value	13.35	56.80	7.28
Alsentzer	short	GAIN-imputation	13.06	57.11	6.70
Huang	short	carry-forward-mean-imputation	12.91	56.93	6.68
Alsentzer	long	GAIN-imputation	12.36	55.72	6.26
Huang	long	GAIN-imputation	12.32	55.17	6.43
Huang	short	distinct-value	12.30	54.91	7.00
Huang	long	carry-forward-mean-imputation	11.69	53.07	6.62

**Table 7:** Evaluation results using *multi-modal* (both *clinical-text* and *non-text*) input features in single-server setting.

clinicalBERT	Text Embeddings Representation	Missingness Representation	AUPRC (%)	AUROC (%)	Earliness (h)
Huang	short	GAIN-imputation	96.55	99.35	4.56
Huang	long	GAIN-imputation	96.09	98.90	4.53
Alsentzer	long	GAIN-imputation	94.04	97.90	4.45
Alsentzer	short	GAIN-imputation	93.95	98.61	4.58
Huang	long	distinct-value	31.95	82.90	3.71
Huang	short	distinct-value	31.31	81.74	3.86
Alsentzer	short	distinct-value	31.06	82.68	3.94
Huang	long	carry-forward-mean-imputation	28.98	81.25	3.87
Huang	short	carry-forward-mean-imputation	28.61	80.57	3.71
Alsentzer	long	carry-forward-mean-imputation	28.37	81.85	3.58
Alsentzer	long	distinct-value	24.33	79.60	3.90
Alsentzer	short	carry-forward-mean-imputation	24.23	77.71	3.98

**Table 8:** Evaluation results using *non-text* input features in a federated learning setting using 10 Raspberry Pi devices.

After Round	Federated Aggregation: Simple			Federated Aggregation: Opt		
	AUPRC (%)	AUROC (%)	Earliness (h)	AUPRC (%)	AUROC (%)	Earliness (h)
1	30.97	86.73	4.00	22.54	82.07	4.01
2	39.77	88.45	4.06	19.78	79.40	3.94
3	8.41	92.80	4.44	18.66	77.86	3.98
4	55.20	95.69	4.53	23.08	81.52	4.04
5	71.39	97.60	4.54	34.83	86.99	4.14

**Table 9:** Evaluation results using *clinical-text* input features in a federated learning setting using 10 Raspberry Pi devices.

After Round	Federated Aggregation: Simple			Federated Aggregation: Opt		
	AUPRC (%)	AUROC (%)	Earliness (h)	AUPRC (%)	AUROC (%)	Earliness (h)
1	13.20	55.60	4.12	12.99	52.06	7.73
2	13.23	54.05	5.63	13.22	51.84	6.63
3	13.79	56.53	6.48	12.85	52.15	6.98
4	13.84	57.52	6.29	13.11	52.93	6.45
5	14.62	58.91	6.30	13.46	54.58	6.85

**Table 10:** Evaluation results using *multi-modal* (both *clinical-text* and *non-text*) input features in a federated learning setting using 10 Raspberry Pi devices.

After Round	Federated Aggregation: Simple			Federated Aggregation: Opt		
	AUPRC (%)	AUROC (%)	Earliness (h)	AUPRC (%)	AUROC (%)	Earliness (h)
1	45.30	93.10	4.47	46.17	93.47	4.43
2	69.26	97.32	4.53	54.66	95.43	4.49
3	60.40	95.73	4.51	42.06	91.89	4.49
4	84.80	98.32	4.54	50.02	94.66	4.52
5	82.44	98.49	4.51	80.58	98.06	4.51

**Table 11:** Evaluation results using *multi-modal* (both *clinical-text* and *non-text*) input features in a federated learning setting using a different number of Raspberry Pi devices.

Total Devices	AUPRC (%)	AUROC (%)	Earliness (h)
**Federated Aggregation: Simple**			
10	82.44	98.49	4.51
9	94.54	99.60	4.53
8	96.13	99.53	4.53
7	97.03	99.63	4.51
6	96.55	99.63	4.51
5	98.79	99.87	4.50
4	98.56	99.76	4.52
3	97.59	99.77	4.50
2	99.20	99.91	4.50
1	99.43	99.94	4.49

**Table 12:** Evaluation results using *non-text* input features in a federated learning setting using three Jetson Nano devices.

After Round	Federated Aggregation: Simple			Federated Aggregation: Opt		
	AUPRC (%)	AUROC (%)	Earliness (h)	AUPRC (%)	AUROC (%)	Earliness (h)
1	40.48	86.10	3.84	38.39	85.46	3.90
2	55.95	95.55	4.50	42.87	87.05	4.04
3	80.42	98.55	4.52	44.31	89.00	4.18
4	92.07	99.30	4.52	48.53	92.03	4.26
5	94.43	99.51	4.53	52.20	93.91	4.30

**Table 13:** Evaluation results using *clinical-text* input features in a federated learning setting using three Jetson Nano devices.

After Round	Federated Aggregation: Simple			Federated Aggregation: Opt		
	AUPRC (%)	AUROC (%)	Earliness (h)	AUPRC (%)	AUROC (%)	Earliness (h)
1	13.31	55.69	5.84	12.79	54.18	7.27
2	15.07	61.73	5.79	13.12	55.65	6.90
3	15.12	63.58	6.43	13.88	57.42	7.06
4	14.33	60.88	6.90	14.14	58.49	7.02
5	14.73	62.24	7.73	14.21	59.72	7.06

**Table 14:** Evaluation results using *multi-modal* (both *clinical-text* and *non-text*) input features in a federated learning setting using three Jetson Nano devices.

After Round	Federated Aggregation: Simple			Federated Aggregation: Opt		
	AUPRC (%)	AUROC (%)	Earliness (h)	AUPRC (%)	AUROC (%)	Earliness (h)
1	94.77	99.34	4.56	94.71	99.44	4.55
2	95.03	99.37	4.54	94.80	99.39	4.56
3	98.07	99.78	4.53	93.58	99.18	4.52
4	98.82	99.87	4.51	95.86	99.48	4.51
5	98.99	99.89	4.51	94.99	99.35	4.52

**Table 15:** Evaluation results using *multi-modal* (both *clinical-text* and *non-text*) input features in a federated learning setting using different number of Jetson Nano devices.

Total Devices	AUPRC (%)	AUROC (%)	Earliness (h)
**Federated Aggregation: Simple**			
3	98.99	99.89	4.51
2	99.36	99.93	4.50
1	99.31	99.92	4.52

## Data Availability

MIMIC-III (Multiparameter Intelligent Monitoring in Intensive Care) database, version 1.4 [32].
